# Protective antigens hidden in the bovine Clostridium vaccine

**DOI:** 10.3389/fimmu.2026.1751922

**Published:** 2026-03-02

**Authors:** He Qin, Jiahui Xie, Qiuyan Deng, Jingjing Ren, Lihe Su, Weiye Zuo, Yue Lv, Haojie Dong, Zhaoquan Liu, Xun Ma, Song Jiang, Jianjun Jiang, Pengyan Wang

**Affiliations:** 1 Laboratory of Animal Immunology Engineering, Department of Animal Medicine, Shihezi University, Shihezi, China; 2 Laboratory of Forage Production and Utilization, Department of Animal Science, Shihezi University, Shihezi, China

**Keywords:** *Clostridium perfringens*, immune efficacy, prokaryotic expression, protective antigen, vaccine

## Abstract

**Background:**

*Clostridium perfringens* type A is the primary pathogenic bacterium responsible for bovine necrotic enteritis, causing significant economic losses in large-scale livestock farming. Our previous reverse vaccinology screen identified the nontoxin antigen protein of this bacterium as highly immunogenic. However, its immune protection efficacy and practical effectiveness in vaccines remain unclear.

**Methods:**

Antigenic epitope analysis, prokaryotic expression, and purification of the *C. perfringens* Plc, ESBP, and YnjE proteins were performed. Mice were immunized with these proteins to detect serum antibody titers, cytokine levels, and changes in the mouse survival rate and weight. The immunoprotective effects of ESBP and YnjE proteins as well as three commercial vaccines were compared by detecting serum antibody titers against the toxin protein Plc and the non-toxin proteins ESBP and YnjE proteins after three immunizations with the vaccines.

**Results:**

Three proteins with high immunogenicity scores (the classic toxin protein Plc and non-toxin proteins ESBP and YnjE) were successfully expressed and purified. ELISA of mouse sera showed significantly elevated levels of IL-2, IL-4, IL-10, and IFN-γ, indicating the induction of a mixed Th1/Th2 immune response. Indirect ELISA confirmed that all three proteins elicited high IgG titers, with the non-toxin ESBP and YnjE inducing higher titers. Their protective efficacy—based on survival rate and body weight recovery—was superior to Vac3, comparable to Vac2, and lower than Vac1. Notably, sera from mice immunized with the three commercial vaccines exhibited low antibody titers against ESBP and YnjE, suggesting that the immunological potential of these two key non-toxin protective antigens is not fully exploited in current vaccines.

**Conclusion:**

ESBP and YnjE exhibited favorable immunogenicity and immune protection in a mouse model. These findings, derived from the mouse model, highlight the great potential of ESBP and YnjE as key candidate antigens for the development of novel vaccines, and provide important experimental evidence for the antigen design and optimization of *C.perfringens* vaccines. These conclusions need to be further validated by in-farm animal trials.

## 1 Introduction

*Clostridium perfringens*, a gram-positive anaerobic bacterium, is ubiquitous in nature and the intestines of animals (1). The toxins produced are classified into seven types (A–G) (2). Type A *C. perfringens* is the most prevalent in cattle disease and is the primary pathogen for hemorrhagic necrotizing enteritis and abomasitis in calves (3, 4). *C. perfringens*-related diseases progress rapidly, and once infection occurs, the cure rate is low, causing significant economic losses for large-scale animal husbandry (3, 5).

Vaccination is one of the most effective measures to prevent *C. perfringens* infection. At present, multivalent inactivated clostridial vaccines are widely used for immunization in farms in Xinjiang Uygur Autonomous Region. These vaccines were originally developed with the core goal of preventing *C. chauvoei* and *C. botulinum* infections. Later, to meet the practical demand of farmers for broad-spectrum prevention and control of clostridial diseases, manufacturers added multiple clostridial antigens such as *C. perfringens* to the original formulation. Although these vaccines have good safety and can provide covering protection against various clostridial diseases, *C. perfringens* is not their core protective target. At present, there are few relevant studies on its actual prevention and control effect against this bacterium, and the real immune protective efficacy lacks clear data support. In addition, with the full implementation of the antibiotic restriction policy in the livestock and poultry breeding field, the use of antibacterial drugs is strictly regulated (6), which has also become an important reason for the frequent occurrence of hemorrhagic necrotic enteritis caused by *C. perfringens* in large-scale farms.

The development of traditional bacterial vaccines relies largely on the strategy of “using toxin proteins as key protective antigens”. This strategy has achieved great success in some vaccines, such as those based on diphtheria toxoid and tetanus toxoid (7). Exert their core mechanism of action by inducing the body to produce specific antibodies that directly neutralize the virulent toxins of pathogens, thereby achieving effective immune protection. However, this toxin-centric research and development model has gradually revealed limitations in combating pathogens with complex pathogenic mechanisms. The pathogenic processes of many important pathogenic bacteria do not depend on a single toxin alone, but involve multiple virulence-related regulatory processes such as intestinal adhesion, *in vivo* colonization and immune evasion. Taking clostridia as an example, its sialidase NanI can participate in the pathogenic process through multiple dimensions by up-regulating the expression of toxins associated with intestinal infection, enhancing the target cell-binding capacity and biological activity of some toxins, and simultaneously promoting the intestinal adhesion ability of the strain itself (8). Type IV pili (TFP) of *Clostridioides difficile* can significantly facilitate the adhesion of the strain to intestinal epithelial cells and contribute to its persistent colonization in the host intestine under the positive regulation of cyclic diguanosine monophosphate (c-di-GMP) (9). In terms of immune evasion, some Gram-positive bacteria can evade the killing and clearance by macrophages. Research on the immune evasion mechanisms of *C. perfringens* is relatively limited, yet the gene encoding lysozyme inhibitor protein(LprI) has been identified in its genome, and the product of this gene is directly associated with the strain’s ability to evade macrophage clearance (10, 11).Against this background, vaccines targeting only a single or multiple toxin antigens can hardly provide comprehensive immune protection for the body.

With the advancement of technologies such as genomics, proteomics and structural biology, researchers have shifted the focus of vaccine development from traditional toxin proteins to exploring nontoxin proteins that play key roles in the life cycle of pathogens, providing a new direction for overcoming the dilemma of traditional vaccine development (12). For example, Rino Rappuoli et al. used reverse vaccinology technology to screen out the key nontoxic antigen proteins fHbp, NHBA and NadA from *Neisseria meningitidis* serogroup B, successfully developed the subunit vaccine Bexsero, and obtained its approval in Europe in 2013 and in the United States in 2015 (13, 14). At present, the immunoprotective effect of nontoxin proteins of *C. perfringens* have been initially verified. Li Zewei et al. reported that the ornithine aminotransferase ArcB and membrane lipoprotein TmpC can induce a strong adaptive immune response in chickens and significantly alleviate the symptoms of necrotic enteritis caused by *C. perfringens* (15). Dwivedi P et al. reported that after mice were immunized with polysaccharide deacetylase family proteins, ABC transporters and lipoproteins, they developed immune protection against *C. perfringens* challenge (16).

The preliminary work of this study was based on the method of reverse vaccinology through whole-genome analysis of 15 strains of pathogenic *C. perfringens* of bovine origin. Based on the proteome encoded by 2304 core genes, two highly conserved surface-exposed non-toxin proteins with excellent immune scores were screened out, namely extracellular solute-binding protein (ESBP) and rhodanese-like domain protein (YnjE) (11). To compare the immunological differences between non-toxin proteins and classical toxin proteins, the classical toxin protein Plc was simultaneously included in this study. The immunogenicity and *in vivo* protective efficacy of the two non-toxin proteins (ESBP and YnjE) and Plc were systematically evaluated through antibody titer detection, challenge protection tests and other assays, so as to clarify the immunological performance of non-toxin proteins in comparison with classical toxin proteins. This study aimed to elaborate on the immunological characteristics of these two non-toxin proteins, verify their potential as vaccine candidate antigens, enrich the antigen library for the development of *C. perfringens* vaccines, and provide new insights and experimental evidence for vaccine research and development.

## 2 Materials and methods

### 2.1 Protein information

Through reverse vaccinology screening, the nucleotide and amino acid sequence information of the following proteins was retrieved from the NCBI and UniProt databases: alpha-toxin, also known as phospholipase C (Plc, NCBI: WP_057230321.1, UniProt: Q1HXB5); extracellular solute-binding protein (ESBP, NCBI: WP_003456161.1, UniProt: A0AAN4CT35); and rhodanese-like domain-containing protein (YnjE, NCBI: WP_195934887.1, UniProt: A0A0H2YSJ8). In addition, a superoxide dismutase protein with weak immunoprotective efficacy (SodF; NCBI: WP_003467108.1; UniProt: A0A0H2YVR5) was identified in preliminary experiments, and its detailed experimental results are provided in **Supplementary Figure 6**. The specific information of all the aforementioned proteins is summarized in **Supplementary Figures S1**-**S3**.

### 2.2 Expression vectors and strains

Type A *C. perfringens* ATCC13124 (accession number: ASM1328v1), the pET-32a plasmid vector, competent *E. coli* DH5α and competent *E. coli* BL21(DE3) cells were preserved in the Animal Immune Engineering Laboratory of Shihezi University.

### 2.3 Experimental animals

Forty 6-week-old female SPF-grade BALB/c mice were purchased from Beijing Sibeifu Bioscience Inc., and 140 6-week-old female clean-grade Kunming mice were obtained from Xinjiang Medical University. All the mice were housed at the Animal Experimental Center of Shihezi University. This animal study was approved by the Animal Ethics Committee of Shihezi University (approval no. A2024–391) and was conducted in accordance with the Guide for the Welfare and Ethics Review of Laboratory Animals.

### 2.4 Key reagents and vaccines

The following key reagents were used Plasmid Mini Kit, Agarose Gel DNA Extraction Kit, and Bacterial Genomic DNA Kit from Beijing TianGen Bio; restriction enzymes (BamHI, EcoRI, and XhoI) and T4 DNA ligase from Takara; LB Broth and Anaerobic Meat Infusion Broth from Qingdao Huabo; BCA Protein Assay Kit, SDS–PAGE Kit, and IPTG from Beijing Solarbio; Ni–NTA agarose from Cytiva; and ELISA Kits for Mouse IL-2, IL-4, IL-10, and IFN-γ from Wuhan Sanying Bio.

The commercial inactivated vaccines used in this study were as follows: Vaccine1, an inactivated multivalent vaccine of *C. chauvoei*, containing *Clostridium septicum*, *C. perfringens* types A–D, *Clostridium sordellii*, *Clostridium novyi*, *Clostridium haemolyticum*, and *Clostridium septicum*; Vaccine2, an inactivated multivalent vaccine of *C. botulinum* type C, such as *C. botulinum* type C and *C. perfringens* types A–D; and Vaccine3, another inactivated multivalent vaccine of *C. septicum*, comprising *C. perfringens* types A–E, *C. novyi*, *C. sordellii*, and *C. haemolyticum*. All the above vaccines use aluminum hydroxide as an adjuvant. Information about the vaccines can be found in the **Supplementary Material**.

### 2.5 Protein antigenic epitope analysis

For B-cell linear epitope prediction, the IEDB tool (http://tools.iedb.org/bcell/) was used, with the BepiPred Linear Epitope Prediction 2.0 option. For B-cell conformational epitope prediction, DiscoTope - 3.0 (https://services.healthtech.dtu.dk/services/DiscoTope-3.0/) was used, with the AlphaFold structure type and moderate confidence options. For T-cell linear epitope prediction, the IEDB tool (http://tools.iedb.org/CD4episcore/) was used, and the IEDB recommended prediction method and a threshold of 50 were selected to obtain epitope scores.

### 2.6 Primer design and synthesis

On the basis of the GenBank sequences of Plc, ESBP, and YnjE, specific primers for expression vector construction were designed using SnapGene software (**Table 1**). The primers were synthesized by Xinjiang Youkang Biotechnology Co., Ltd.

**Table 1:** Target gene specific primers and vector validation primers.

Primer	Sequence (5’-3’)	Fragment size
CPF_Plc E-F	CCG**GAATTC**ATGAAAAGAAAGATTTGT	1215 bp
CPF_Plc X-R	CCG**CTCGAG**TTTTATATTATAAGTTGAATTTC	
CPF_ESBP B-F	CGC**GGATCC**ATGAGAAAAAAAATTCTAAGT	1278 bp
CPF_ESBP X-R	CCG**CTCGAG**TTTATTTATAAGTTCACTTG	
CPF_YnjE B-F	CGC**GGATCC**ATGAGTTTATTAAAAAAATTAA	1374 bp
CPF_YnjE X-R	CCG**CTCGAG**CTATTTTGTAGGCTCCTT	
pET-32a validation	F:GGATGAAATCGCTGACGA	empty vector
primer	R:AACTCAGCTTCCTTTCGG	466 bp

The bold characters indicate the restriction enzyme cleavage sites in the primers.

### 2.7 Vector construction, induction, and purification

Three target genes were amplified by PCR using the primers in section 2.6. The products were detected by 1% agarose gel electrophoresis and then recovered. The target genes and pET-32a plasmid were subsequently subjected to double enzyme digestion, followed by ligation and transformation into *E. coli* DH5α competent cells. After incubation at 37 °C for 12 h, single colonies were picked and cultured, and PCR verification was performed using the target gene primers and 32a verification primers listed in Table 3-1. The pET-32a-target gene plasmids were extracted using a plasmid miniprep kit and sent to Xinjiang Youkang Biotechnology Co., Ltd. for sequencing.

The recombinant plasmids were transformed into the expression strain BL21 to construct the expression strains BL21-32a-Plc, BL21-32a-ESBP, and BL21-32a-YnjE. These expression strains were inoculated into LB liquid medium and induced for expression with 1 mM IPTG for 8 h. After repeated freeze-thawing with liquid nitrogen, ultrasonic disruption was performed. ESBP protein was expressed in a soluble form, whereas Plc and YnjE proteins were expressed as inclusion bodies. Plc, ESBP, and YnjE proteins were obtained after Ni column affinity chromatography, dialysis, and concentration. The protein concentrations were determined, and the proteins were stored at -80 °C for later use.

### 2.8 Immunization and challenge experiments

Animal immunization and challenge experiments in this study were conducted in two phases using different mouse models: The preliminary Verification phase employed inbred BALB/c mice. This strain has a homogeneous genetic background, which can effectively minimize the interference of individual differences on experimental results, and was used to evaluate the immunogenicity and protective efficacy of recombinant proteins. The extended comparison phase adopted outbred Kunming mice, which can reflect population-level immune response differences and are suitable for comparing the protective efficacy of recombinant proteins and commercial vaccines.

#### 2.8.1 Preliminary verification phase (BALB/c mouse model)

Forty female BALB/c mice were divided into four groups, with 10 mice per group, namely the PBS control group, Plc toxin protein group, ESBP non-toxin protein group and YnjE non-toxin protein group. The mice in the control group were intraperitoneally injected with 150 μL of PBS solution. In the Plc, ESBP, and YnjE groups were intraperitoneally injected with 150 μL of the corresponding protein solution diluted in PBS, containing 100 μg of target protein each. Immunization was performed via intraperitoneal injection, with three immunizations spaced two weeks apart. Blood was collected one week after each immunization to separate the serum, and this process was repeated three times. Ten days after the third immunization, the mice in each group were intraperitoneally injected with 0.15 mL of ATCC13124 bacterial suspension in PBS at the median lethal dose (LD_50_ = 2.068×10^8^ CFU/mL). Mortality and body weight changes of the mice were recorded continuously for 7 days post-challenge.

#### 2.8.2 Extended comparison phase (Kunming mouse model)

To investigate the differences in immune protective efficacy between the superior-performing non-toxin protein groups (ESBP and YnjE) and the commercial vaccine group, the challenge dose was increased.However, high-dose challenge may lead to complete mortality of mice in the PBS control group within the first two days; thus, a continuous control for the mental state and weight changes of the mice is lacking. Therefore, a SodF protein group was added as an auxiliary control group in the immunization and challenge process because of its weak immunoprotective effect. Plc group was omitted in this phase since its protective efficacy was already confirmed to be significantly weaker than that of ESBP and YnjE in the preliminary phase. First, 140 Kunming mice were divided into seven groups, with 20 mice per group. The groups were as follows: a PBS control group, a weak immunoprotective protein SodF group, recombinant protein ESBP and YnjE groups, and commercial vaccine groups (Vac1, Vac2, and Vac3). Each mouse in the control group was intraperitoneally injected with 150 μL of PBS solution. Mice in the ESBP group were injected with 150 μL of ESBP protein solution diluted in PBS, containing 100 μg of protein. Similarly, mice in the SodF and YnjE groups were injected with 150 μL of protein diluent containing 100 μg of protein. The dosage for the vaccine groups (Vac1, Vac2, and Vac3) was unified at 150 μL. The immunization method was the same as that for the protein groups, with intraperitoneal injection. Both the vaccine and protein groups were immunized three times with a two-week interval between each immunization. One week after the third immunization, blood was collected from the mice in each group to separate the serum, and this was performed once. Ten days after the third immunization, each group of mice was intraperitoneally injected with 0.15 mL of ATCC13124 bacterial suspension in PBS at the absolute lethal dose (LD_100_ = 2.5×10^10^ CFU/mL, actual CFU per mouse = 3.75×10^9^). Specific data are shown in **Supplementary Tables S2** and **S3** in the **Supplementary Material**. During the experiment, when the mice met the preset moribund criteria (weight loss>20%, activity score ≤ 1 point), they were immediately euthanized by cervical dislocation after inhalation anesthesia to reduce suffering, and the death time and weight changes were recorded. Mice surviving after challenge continued to be raised.

### 2.9 Detection of mouse serum IgG antibody levels

An indirect ELISA established in our laboratory was used to detect mouse serum antibodies. BALB/C mice were immunized three times. One week after each immunization, blood was collected, allowed to stand at 4°C for 2 h, and centrifuged at 3000×g for 10 min to separate the serum. Kunming mice were immunized three times with the Vac1, Vac2, and Vac3 vaccines; serum was collected after the third immunization and stored at -40°C.

Plc, ESBP, and YnjE proteins were diluted to 10 µg/mL with carbonate buffer (pH=9.6), added to a 96-well plate (100 µL per well), and coated overnight at 4°C. After the samples were washed 3 times with PBST, 200 µL of 5% nonfat milk was added to each well, and the samples were blocked at 37°C for 2 h. Following 3 washes with PBST, 100 µL of mouse serum (serially diluted starting from 1:400) was added to each well and incubated at 37°C for 1 h. After 3 additional washes with PBST, 100 µL of HRP-labeled goat anti-mouse IgG (diluted 1:4000) was added to each well and incubated at 37°C for 1 h. Then, after 3 additional washes with PBST, 100 µL of TMB single-component chromogenic solution was added to each well and the samples were allowed to react at room temperature for 10 min. The reaction was stopped by adding 100 µL of 1 mol/L sulfuric acid solution to each well. The OD450 was measured using a microplate reader.The cutoff value was 2.1 times that of negative serum. When the P/N ratio was greater than 2.1, the sample was judged as positive, and the corresponding maximum dilution factor was determined as the antibody titer.

### 2.10 Cytokine quantification

To compare the differences in cytokine levels induced by the three proteins in infected mice, blood samples were collected one week after the second immunization. The concentrations of cytokines(IL-2, IL-4, IL-10, and IFN-γ) were measured using sandwich ELISA kits.

### 2.11 Assessment of immunoprotective efficacy

Ten days after the final immunization, the mice were challenged with the ATCC13124 strain. Two hours before challenge, sufficient food and water were provided to prevent weight measurement errors. The mice were monitored for food intake, water intake and general condition, and adequate nutrition was maintained throughout. Body weight was measured and recorded, and changes in body weight were monitored over 7 days after the challenge. The survival rate was calculated to evaluate the immunoprotection of the antigenic proteins via the following formula:

immunoprotection rate (%) = [(number of deaths in the control group − number of deaths in the immunized group)/number of deaths in the control group] × 100%.

### 2.12 Data analysis

Data processing and analysis were performed using multiple software platforms. Preliminary data organization and summarization were conducted in Excel 2016. Statistical analyses, including one-way ANOVA, proportional analyses, and two-way analysis of variance (ANOVA) followed by Tukey’s honestly significant difference (HSD) *post-hoc* test for multiple pairwise comparisons, were carried out using SPSS 26.0.

## 3 Results

### 3.1 Antigenic epitope analysis of three proteins

**Figure 1:**
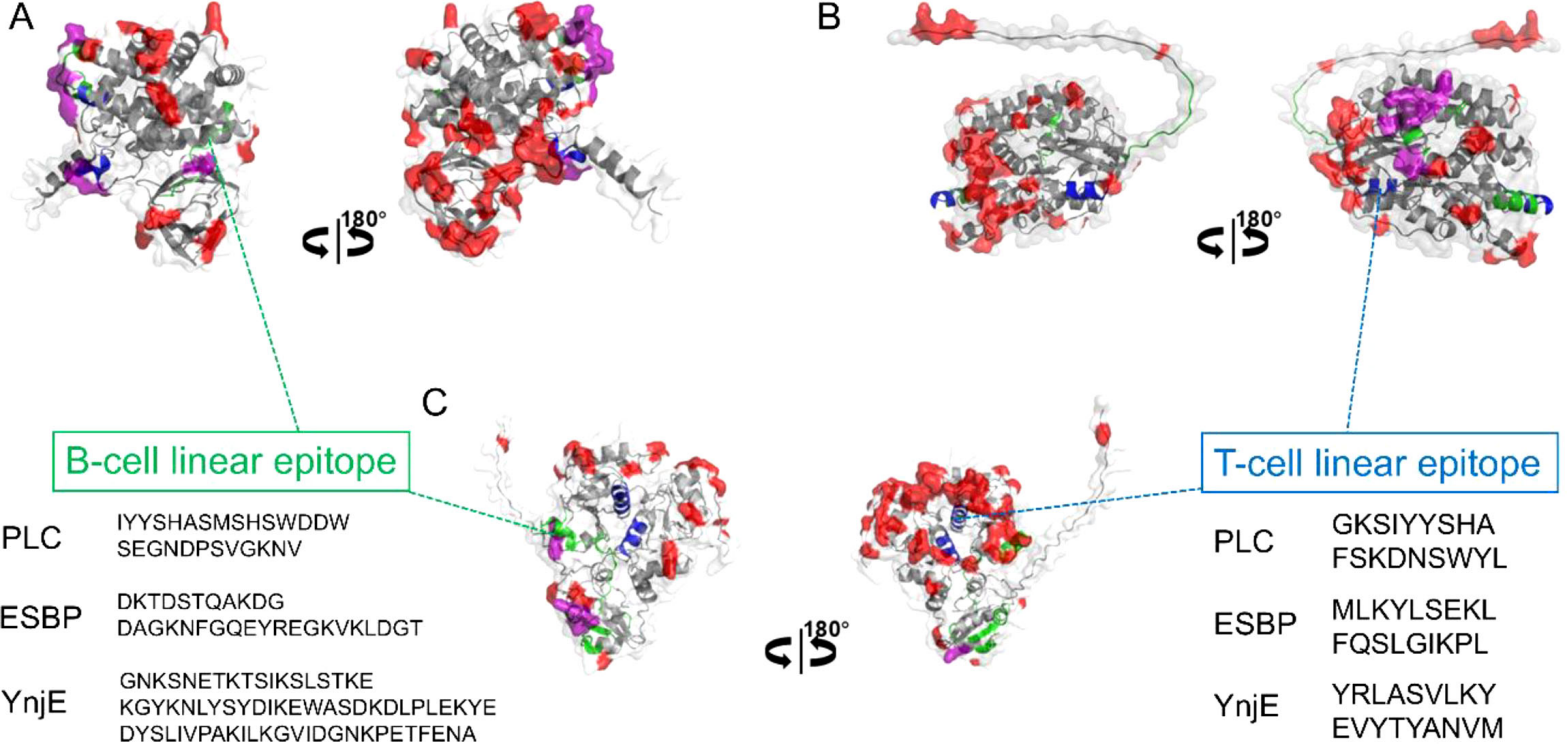
Mappin of predicted B-cell and T-cell epitopes on the tertiary structures of candidate antigenic proteins. **(A)** Plc protein. **(B)** ESBP protein. **(C)** YnjE protein. The three-dimensional protein structures are depicted in light gray cartoon representation. Predicted linear B-cell and T-cell epitopes are mapped onto the structures and highlighted in green and blue, respectively. The top four highest-scoring linear B-cell epitopes are labeled for each protein. Predicted conformational B-cell epitopes on the protein surface are colored in red. Regions where linear and conformational epitopes overlap are shown in purple. This structural mapping demonstrates that the candidate proteins possess multiple high-scoring, surface-exposed epitopes, supporting their strong potential for eliciting robust immune responses.

Through epitope prediction analysis, we obtained both the scoring values and precise locations of the epitopes on the target proteins. Using PyMOL software, we mapped the top four linear B-cell epitopes and high-scoring conformational epitopes onto the three-dimensional structure of each protein (**Figure 1**). Among the Plc, ESBP and YnjE proteins, all the predicted T-cell epitopes scored above 90, with the YRLASVLKY epitope of YnjE achieving the highest score of 95.7. With respect to B-cell epitopes, the SEGNDPSVGKNV epitope of Plc received the highest score. Both the toxin and non-toxin proteins showed high immunogenicity scores, which indicated their favorable immunogenic potential and justified further research and development.

### 3.2 Expression and Western blotting of target proteins

**Figure 2:**
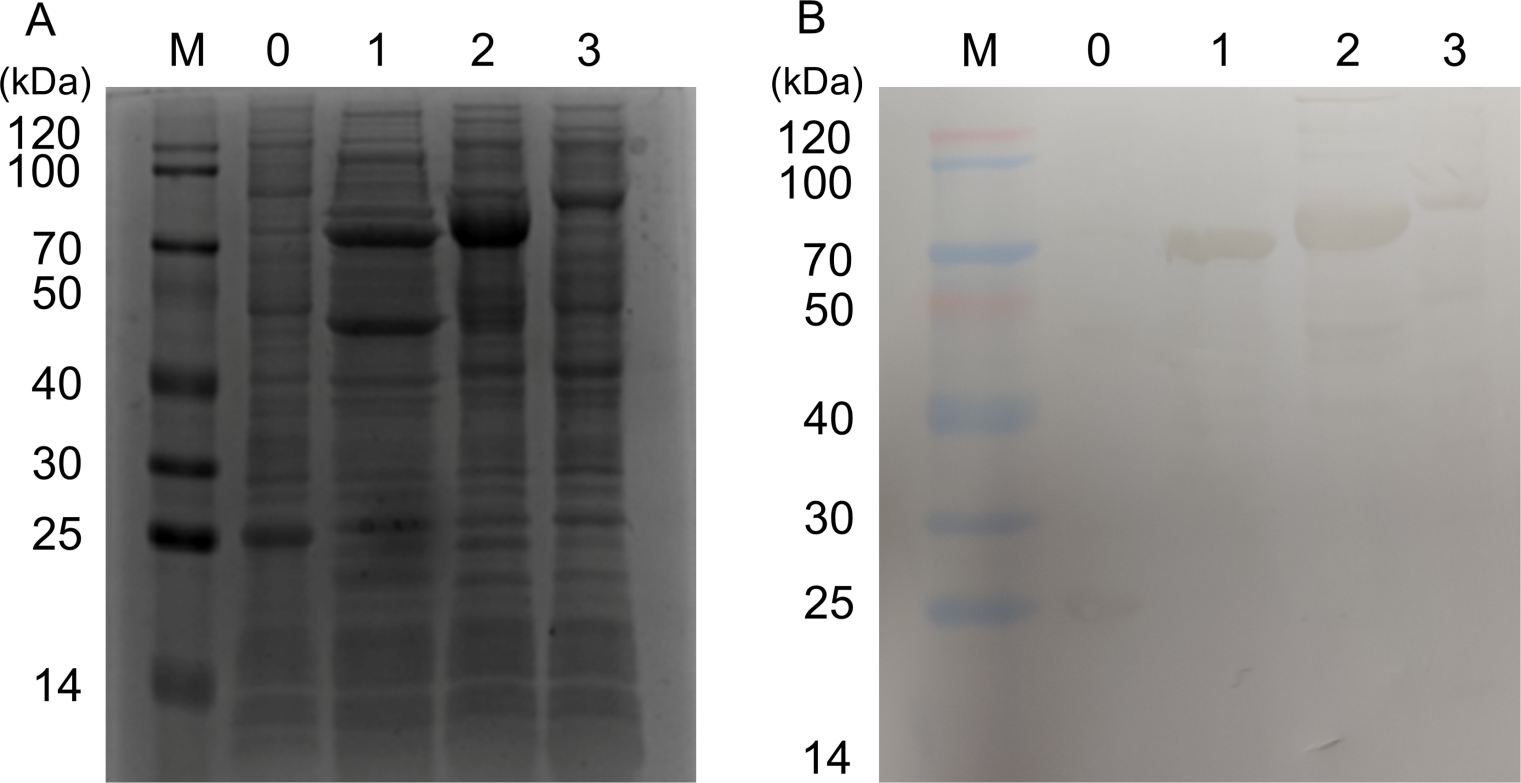
Expression and Western blot verification of recombinant Plc, ESBP, and YnjE proteins in *E. coli*. **(A)** SDS-PAGE analysis of the expressed recombinant proteins. Total proteins from *E. coli* lysates were separated by SDS-PAGE after induction with 1 mM IPTG. The predicted molecular weights for the pET-32a vector tag, and the recombinant pET-32a-Plc, pET-32a-ESBP, and pET-32a-YnjE fusion proteins are approximately 25 kDa, 70.5 kDa, 72.3 kDa, and 74.9 kDa, respectively. **(B)** Western blot analysis confirming the identity of the expressed proteins. The proteins from **(A)** were probed with an anti-His tag mouse monoclonal primary antibody. Specific bands detected at the expected molecular sizes confirm the successful expression of the His-tagged recombinant proteins. Lane M: Protein molecular weight marker; Lane 0: pET-32a empty vector control; Lane 1: pET-32a-Plc; Lane 2: pET-32a-ESBP; Lane 3: pET-32a-YnjE.

To verify protein expression, the successfully constructed recombinant plasmids were transformed into *Escherichia coli* (*E. coli*) and induced with IPTG. Bacterial cells were harvested and ultrasonically lysed, and the lysate samples were analyzed by SDS-PAGE. As shown in **Figure 2A**, the protein band sizes of the empty pET-32a vector control group and the pET-32a-Plc, pET-32a-ESBP, and pET-32a-YnjE experimental groups were approximately 25 kDa, 70.5 kDa, 72.3 kDa, and 74.9 kDa, respectively, which were consistent with the expected molecular weights of each recombinant protein. This finding preliminarily indicated the successful expression of the target proteins.

Further verification was performed by Western blotting. Detection was carried out using mouse anti-His tag IgG as the primary antibody, and the results revealed that specific bands appeared in each experimental group at positions corresponding to their expected molecular weights (**Figure 2B**). These results indicated that the recombinant Plc, ESBP, and YnjE proteins were all successfully expressed in the prokaryotic system.

### 3.3 Recombinant protein purification

**Figure 3:**
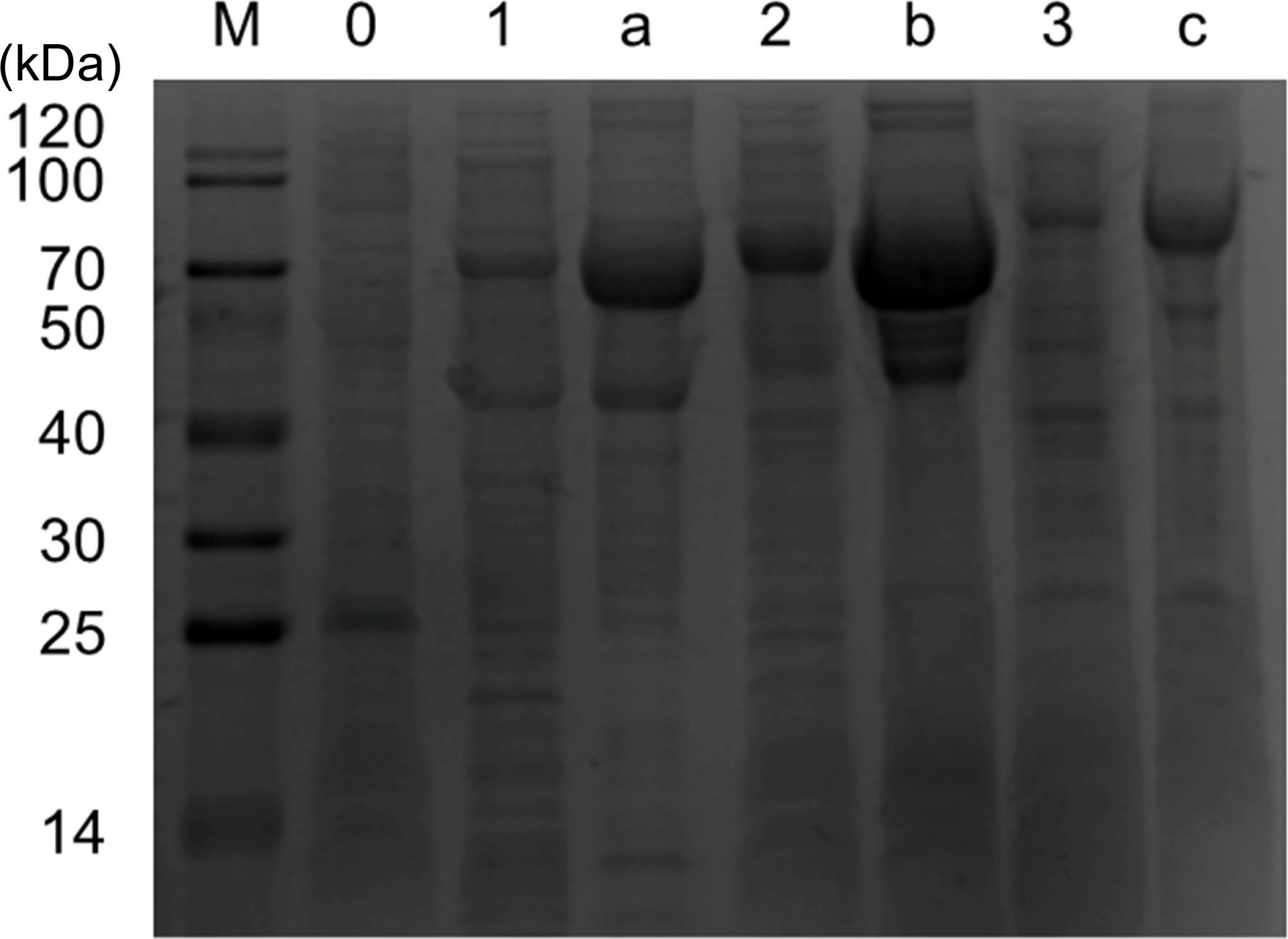
SDS-PAGE analysis of protein expression and purification. Total cell lysates (Lanes 1-3) and the corresponding purified proteins (Lanes a-c) for Plc, ESBP, and YnjE were analyzed. Lanes 0 and M represent the empty vector control and protein marker, respectively. The purified fractions (Lanes a-c) show prominent bands at the expected sizes with minimal amounts of contaminating proteins, confirming the effectiveness of the Ni²⁺ affinity chromatography-based purification procedure.

After the expression of the recombinant proteins was induced for with 1 mM IPTG for 8 h, the proteins were purified by nickel ion affinity chromatography, desalted via dialysis and concentrated through ultrafiltration. The purified and concentrated proteins, together with the total bacterial proteins after IPTG induction, were subjected to simultaneous SDS-PAGE analysis (**Figure 3**). The results revealed that in Lanes 1, 2, and 3 (postinduction), although target protein bands could be observed, they were accompanied by multiple bands of contaminating proteins, indicating low purity of the target proteins. In Lanes a, b, and c (after purification and concentration), the target protein bands were clear, with their corresponding molecular weights consistent with expectations; the bands of contaminating proteins were significantly reduced. Visually, the proportion of contaminating proteins greatly decreased, whereas the proportion of target protein bands significantly increased.

These results indicate that the purification strategy employed in this study—combining nickel ion affinity chromatography with dialysis and ultrafiltration concentration—can effectively remove contaminating proteins, ultimately enabling the stable acquisition of high-purity recombinant proteins, thus laying a foundation for subsequent experiments.

### 3.4 Indirect ELISA for serum-specific antibody detection

**Figure 4:**
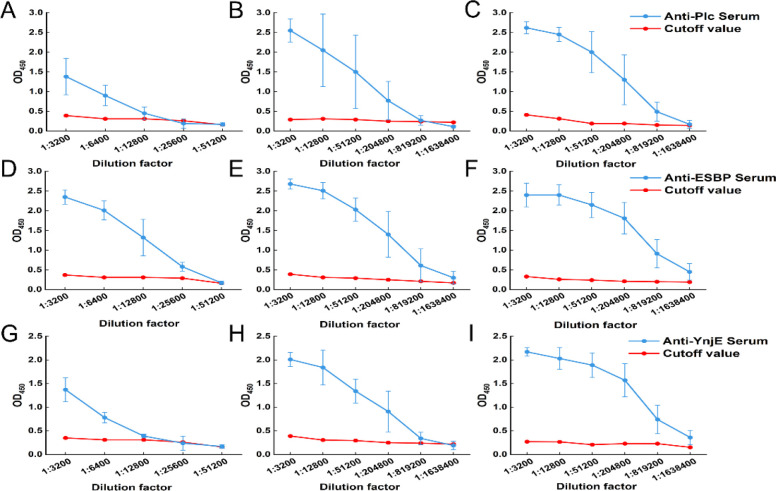
Sequential monitoring of polyclonal antibody titers following immunization. Indirect ELISA analysis of serum antibody titers specific for Plc **(A-C)**, ESBP **(D-F)**, and YnjE **(G-I)** proteins after each of the three immunizations. The red line indicates the assay cutoff value. The results demonstrate a potent and boosting effect of immunization with all three candidates, leading to a massive increase in antigen-specific antibody titers, confirming their strong immunogenicity.

To monitor the efficiency of the humoral immune responses induced by the recombinant proteins, tail tip blood was collected from the mice on day 7 after each immunization. The blood was allowed to stand at 4°C to separate the serum, and indirect ELISA was then used to detect the specific antibody titers against the three recombinant proteins (Plc, ESBP, and YnjE) in the serum (**Figure 4**). Serum from nonimmunized mice was used as the negative control, and 2.1 times the OD_450_ nm value of this control was set as the cutoff value to eliminate background interference. If the OD value of the serum to be tested at a certain dilution was greater than or equal to this standard, the serum was considered positive, and the corresponding maximum dilution factor was the antibody titer. After the first immunization, the antibody titers of Plc, ESBP, and YnjE were 1:6400, 1:25600, and 1:6400, respectively, with their corresponding OD values all significantly higher than the cutoff value. These findings indicated that all three proteins could effectively initiate humoral immune responses in mice, among which ESBP had a slightly superior initial triggering ability. After the second immunization, the antibody titers of the three proteins increased by dozens of times. Plc, ESBP, and YnjE increased to 1:204800, 1:819200, and 1:409600, with OD values still stably higher than the cutoff value. These findings confirmed that booster immunization could significantly increase the production efficiency of specific antibodies. After the third immunization, the antibody titers further increased and plateaued. The final titers of Plc, ESBP, and YnjE were 1:819200, 1:1638400, and 1:1638400, respectively, which were 128-fold, 64-fold, and 256-fold higher than those after the first immunization, respectively. Moreover, the OD value at the maximum dilution still met the positive standard, indicating that the antibody secretion level reached a high level with stable specificity.

In summary, the three recombinant proteins (Plc, ESBP, and YnjE) all effectively induced specific humoral immune responses in mice. After three immunizations, the antibody titers reached a high level and remained stable. Among them, the YnjE protein showed the greatest increase in titer, whereas the initial triggering ability of the ESBP protein was better.

### 3.5 Cytokine levels in mouse sera after immunization

**Figure 5:**
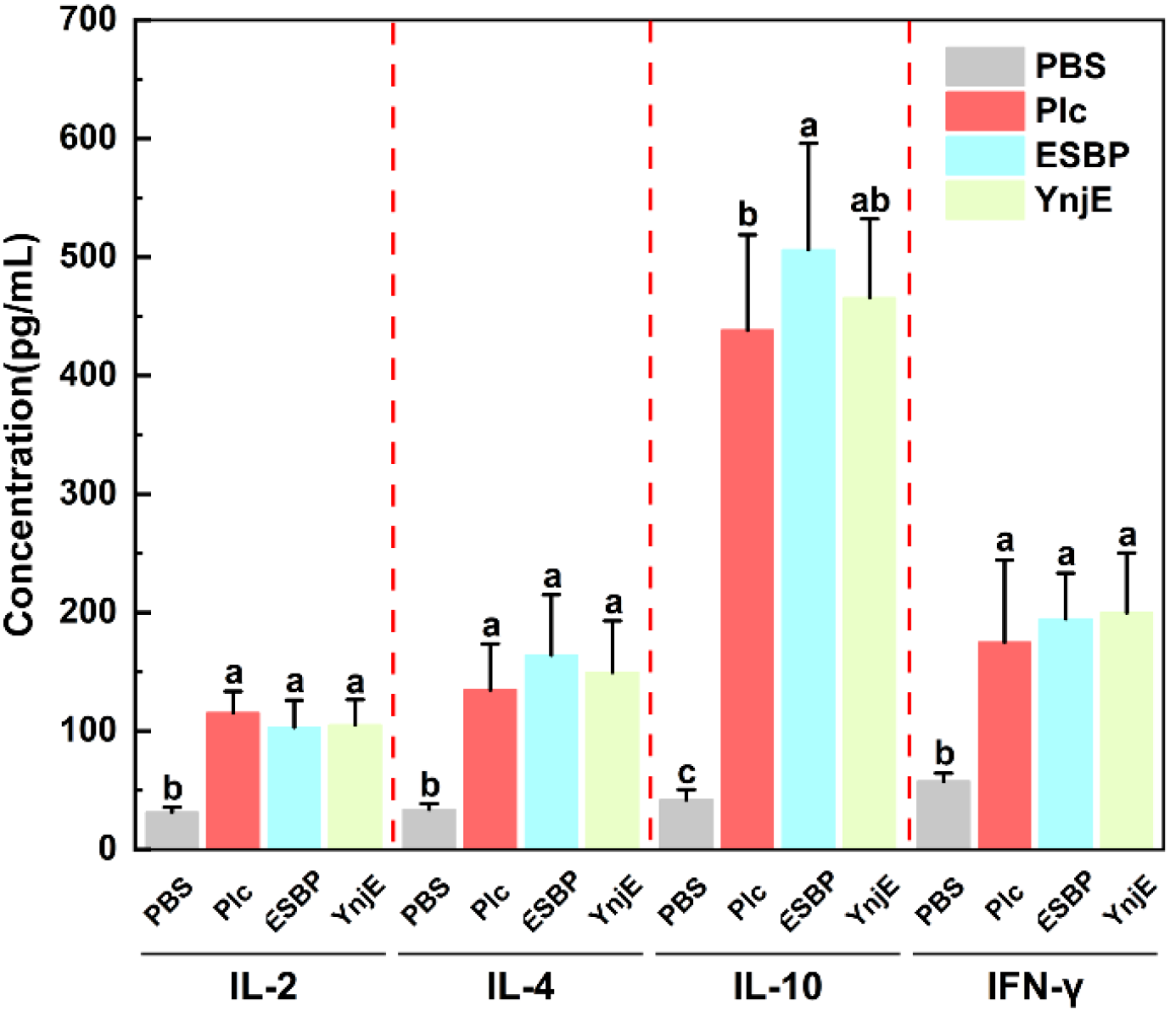
Detection of serum cytokines. Serum concentrations of IL-2, IL-4, IL-10, and IFN-γ were quantified by ELISA after the second immunization with Plc, ESBP, or YnjE protein. Values marked with different lowercase letters (a, b, and c) for the same cytokine denote statistically significant differences (p < 0.05). Compared with the PBS control group, all immunized groups exhibited significantly elevated levels of all detected cytokines; notably, IL-4 and IL-10 were highly induced, indicative of a mixed Th1/Th2 immune response with a Th2 polarization.

To compare the cytokine levels induced by the classical toxin protein (Plc) and two non-toxin proteins (ESBP and YnjE) in infected mice, the IL-2, IL-4, IL-10, and IFN-γ levels in mouse serum were detected one week after the second immunization using indirect ELISA (**Figure 5**). Compared with those in the PBS group, the protein immunized groups presented significantly higher levels of IL-2, IL-4, and IFN-γ (P < 0.05). Compared with the Plc group, the ESBP group had notably higher IL-10 levels (P < 0.05), and the IL-10 levels did not significantly differ between the Plc and YnjE groups (P > 0.05). As a pleiotropic anti- inflammatory cytokine produced by macrophages, Tregs, and Th2 cells, IL-10 suppresses Th1 type immune responses and reduces inflammation. IL-4 and IL-10 are key Th2 type immune response cytokines. These results indicated that all three proteins effectively stimulated the secretion of both Th1-type (IL-2, IFN-γ) and Th2-type (IL-4, IL-10) cytokines in mouse serum, with the latter being predominant in the cytokine profile. This finding demonstrates that immunization with the recombinant proteins elicited a mixed Th1/Th2 immune response with a distinct Th2 bias.

### 3.6 Assessment of immunoprotection

**Figure 6:**
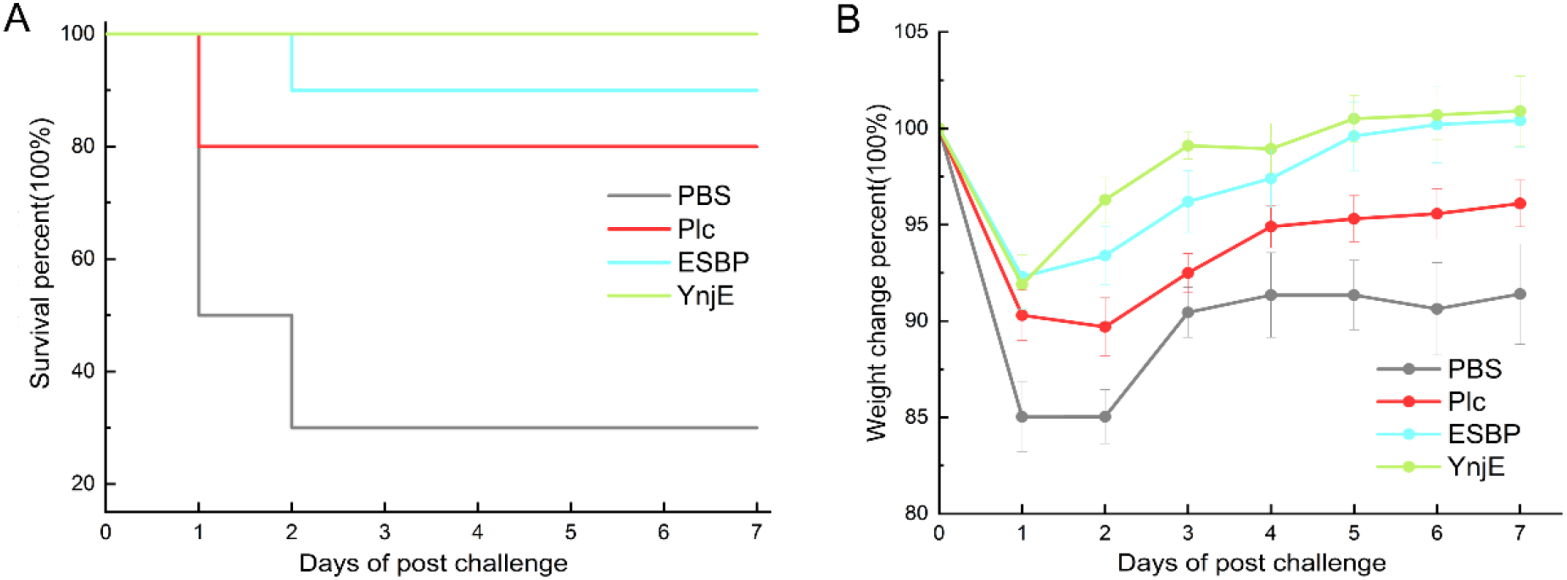
Survival and body weight changes following pathogen challenge. **(A)** Survival curves and **(B)** daily body weight changes (as a percentage of initial weight) in immunized mice after challenge. Compared with the PBS control, all the recombinant proteins significantly improved survival and mitigated weight loss. A hierarchy of protective efficacy was established: YnjE (100% survival, fastest weight recovery) > ESBP (90% survival) > Plc (80% survival). The poorest outcomes were observed in the PBS group (30% survival and persistent weight deficit).

To evaluate the protection rate of the proteins, the mice were challenged via intraperitoneal injection, and the survival and weight changes were monitored for one week (**Figure 6**). Survival curves revealed that most deaths in the PBS, Plc, ESBP, and YnjE groups occurred within the first two days, especially on the first day. No deaths occurred from day 3 onward. The 7-day survival rates were 30%, 80%, 90%, and 100% for the PBS, Plc, ESBP, and YnjE groups, respectively. In terms of weight changes, the greatest loss occurred on day 1 after the challenge, with the greatest intergroup differences occurring on day 2(two-way ANOVA with Tukey’s *post-hoc* test, *P* < 0.05). On day 1, the mice in the PBS group lost 15% of their body weight, whereas the mice in the ESBP and YnjE groups lost 7.7–8.1% of their body weight(*P* < 0.01 vs. PBS group). On day 2, the body weight lost percentages of the YnjE and ESBP groups were 4.4% and 1.1%, respectively. The Plc group continued to lose weight, with an average loss of 0.6% (cumulative 10.3%, *P* < 0.05 vs. ESBP/YnjE groups), whereas the weight of the PBS group remained at 15% below the initial weight (*P* < 0.01 vs. all protein-treated groups). On day 3, the surviving mice in all the groups showed rapid weight recovery(*P* < 0.05 vs. day 2 for all groups). In the PBS group, the maximum body weight gain reached 5.4%, and the average body weight was ultimately restored to 90.5% of the original The weights of the mice in the YnjE and ESBP groups returned to their initial weights by days 5 and 6, respectively, whereas those of the mice in the Plc and PBS groups did not return to their initial body weights by day 7. The immunoprotection rates were 71.4%, 85.7%, and 100% for the Plc, ESBP, and YnjE groups, respectively. The weight change data demonstrated that the two non-toxin proteins (ESBP and YnjE) exerted better immune protective effects than the toxin protein (Plc), with YnjE exhibiting the most prominent protective efficacy.

### 3.7 Comparison of vaccine and protein immunization effects

**Figure 7:**
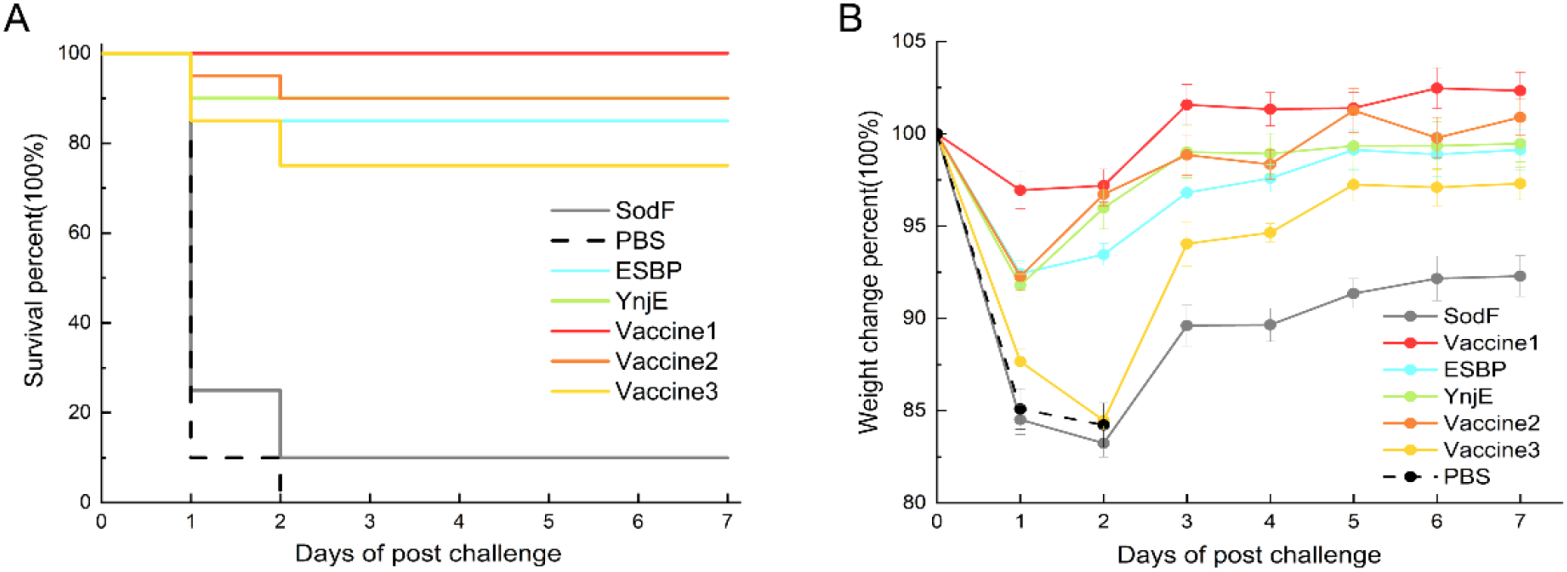
Survival and body weight changes following pathogen challenge. **(A)** Survival rates over 7 days after challenge. **(B)** Dynamic body weight changes are presented as a percentage of the initial weight. The PBS and SodF groups had the poorest outcomes, with complete or near-complete mortality and significant weight loss. In contrast, the ESBP and YnjE proteins conferred strong protection, with survival rates and weight recovery profiles comparable to those of the Vac2 vaccine group.

According to the survival curve (**Figure 7A**), all the PBS-treated mice died within the first two days after the challenge, with mortality rates similar to those of the SodF group, which had a final survival rate of 10%. By contrast The ESBP, YnjE, Vac1, Vac2 and Vac3 groups presented comparable survival rates of 75–100% within seven days after the challenge. Among the vaccine groups, the survival rate of the Vac1 group was the highest at 100%, whereas that of the Vac3 group was the lowest at 75%. The survival rates of the ESBP and YnjE groups were 85% and 90%, respectively. According to the results of the weight-change analysis (**Figure 7B**), the weight loss of the Vac3 and SodF groups continued during the first two days, and the SodF group experienced the greatest weight loss of 17.3% and failed to recover to its initial weight within seven days. The average weight of the Vac1 group was greatest, with the least weight loss on day 1 and recovery to above the initial weight by day 3 (*P* < 0.01 vs. SodF groups on day 3). The weight loss of the ESBP, YnjE and Vac2 groups was significant on day 1 (*P* < 0.05 vs. Vac1 group) but gradually recovered between days 2 and 7. The calculations revealed that the immunoprotection rate of the Vac1 group was the highest at 100%, whereas that of the Vac3 group was the lowest at 75%. Among the protein groups, the YnjE group had the highest immunoprotection rate at 90%, followed by the ESBP group at 85% and the SodF group at just 10%. The protective efficacy evaluated based on survival rate and body weight changes showed that in the intraperitoneal challenge model, the SodF protein induced weak protective effects, with the survival rate of mice as low as 30%. In contrast, the YnjE and ESBP proteins exhibited stronger protective efficacy—while their protective levels were lower than those of Vac1, they were comparable to Vac2 and superior to Vac3.

### 3.8 Specific antibody levels against four proteins in sera from commercial vaccine-immunized mice

**Figure 8:**
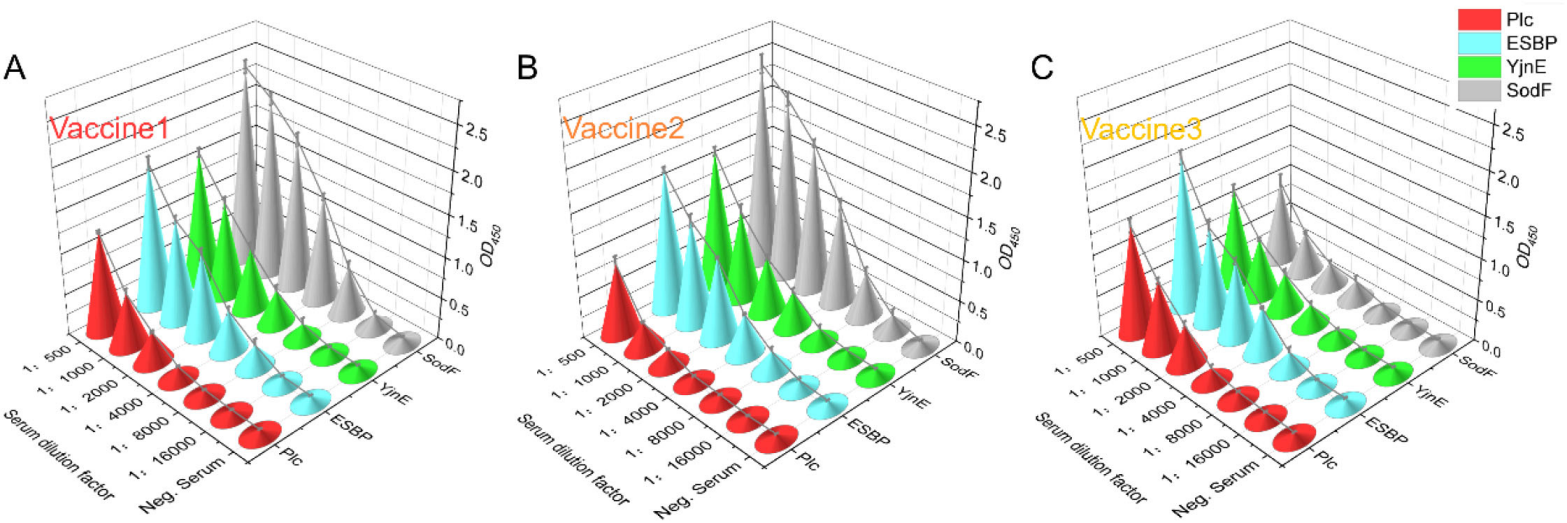
Serum antibody titers against specific vaccine components have a limited association with the protective outcome. **(A–C)** Measurements of antigen-specific antibody responses in mice immunized with three commercial vaccines (V1-V3) showed that all vaccines elicited specific antibodies against the four proteins, but the antibody titers were generally low. No significant difference was observed between the Vac1 and Vac2 groups, whereas both groups exhibited significantly higher antibody titers against YnjE and SodF proteins compared with the Vac3 group (P < 0.05).

To investigate the serum antibody levels induced by four proteins (Plc, ESBP, YnjE, and SodF) in mice immunized with commercial vaccines, an indirect ELISA was performed using the mouse sera from Vac1, Vac2 and Vac3 immunized groups obtained as described in Section 2.8.2. Specific antibody titers against these four proteins were detected with their corresponding purified recombinant proteins as coating antigens (**Figure 8**). The results showed that although all four proteins were present in the commercial inactivated vaccines, the overall titers of the induced specific antibodies were relatively low, with the highest titer (against the SodF protein) only reaching 1:16,000.

Further comparison of antibody responses among different commercial vaccine groups revealed the following results: the antibody titers against the four proteins in mice immunized with Vac1 (exhibiting the best immunoprotective efficacy) were basically consistent with those in mice immunized with Vac2 (with slightly inferior efficacy), which were 1:2,000 for Plc, 1:8,000 for ESBP, 1:8,000 for YnjE, and 1:16,000 for SodF, respectively. In contrast, when comparing Vac1 with Vac3 (with poor protective efficacy), there were no significant differences in the specific antibody titers against Plc and ESBP proteins, and the only difference was observed in the antibody titer against YnjE protein (1:8,000 for Vac1 vs. 1:4,000 for Vac3).

In conclusion, although all four antigens were present in the commercial vaccines and could induce the body to produce specific antibodies, their overall antibody levels were relatively low, and significantly lower than those induced by the separate immunization with each corresponding recombinant protein (P<0.05).

## 4 Discussion

### 4.1 Epitopes are key determinants of protein immunogenicity

In this study, three proteins—the Plc toxin protein and the ESBP and YnjE nontoxin proteins—all of which are surface-exposed proteins, including secreted proteins and cell wall-associated proteins, were investigated. Unlike cytoplasmic proteins localized inside bacteria, which require antigen-presenting cells (APCs) to internalize, process, and present antigenic peptides (linear B-cell epitopes) via MHC class II molecules for recognition by CD4+ T cells, surface-exposed proteins can be directly recognized by B-cell receptors (BCRs) through their conformational epitopes, in addition to being processed into linear epitopes by APCs. From this perspective, surface-exposed proteins exhibit greater accessibility, making them more likely to elicit robust immune responses.

The accuracy of epitope prediction has long been a subject of debate (17). Although theoretically any region on the surface of an antigen can bind antibodies, certain areas are inherently more prone to being targeted by the immune system (**Figure 1**, purple regions) and are thus more immunogenic. This phenomenon is termed immunodominant epitopes (18). Therefore, predicting epitopes and selecting proteins enriched with immunodominant epitopes for experimental studies represents an efficient and effective strategy.

### 4.2 Immunization with recombinant proteins

The “messenger molecules” of the immune system are cytokines. Determining cytokine levels is highly important for understanding the type of immune response activation and evaluating its protective efficacy. IL-2 and IFN-γ are key cytokines involved in Th1 type immune responses, whose main function is to activate cellular immunity to combat intracellular pathogens and tumor cells (19). The key cytokines involved in Th2 type immune responses are IL-4 and IL-10, which mainly activate humoral immunity and form a complementary and dynamic balance with Th1 type immune responses (20, 21). This study found that the immune response induced by the recombinant proteins was a mixed Th1/Th2 type with a distinct Th2 bias, highly consistent with the feature that *C. perfringens* infects the host via exotoxins and elicits a predominantly Th2-type host immune response.

In selecting experimental animals, several practical constraints such as the high cost and operational challenges associated with bovine studies, led us to employ BALB/c mice and Kunming mice as surrogate models. BALB/c mice are a well-established model for investigating the immunoprotective efficacy against *C. perfringens* (22–24). Owing to their affordability and wide availability, KM mice have also been extensively used in studies assessing bacterial pathogenicity, immunogenicity, and model construction, yielding reliable results (21, 25).

As key components of vaccines, the scientific use of adjuvants can significantly enhance the immune effect of antigens (26). However, during the early mechanistic validation phase of this study, the use of Freund’s adjuvant resulted in local induration and antibody titer fluctuation. Furthermore, such potent adjuvants may mask the genuine differences in immunogenicity and protective efficacy among different antigens (27). Therefore, to objectively evaluate and compare the intrinsic immunogenicity of each recombinant protein, the present study used antigen proteins diluted in PBS for immunization, so as to more accurately reveal the intrinsic immune potential of the recombinant proteins.

High-titer specific IgG (1:1,600,000) was detected in inbred BALB/c mice during the preliminary stage of this study, which was closely related to their homogeneous genetic background, multiple booster immunizations (28, 29), the intrinsic immunogenicity of the high-purity recombinant proteins, and blood collection at the peak time point of IgG antibody levels (30, 31). This high titer only appeared in inbred mice during the preliminary antigen screening stage, while the antibody titer was significantly decreased in outbred Kunming mice in the subsequent stage, which is consistent with the different application orientations of the two mouse models in this study.

For the challenge experiment, intraperitoneal injection was chosen as the route of administration. Although type A *C. perfringens* causes primarily bovine gastrointestinal diseases such as hemorrhagic necrotic enteritis and abomasitis, mice are not naturally susceptible to this pathogen. Although oral challenge can induce intestinal symptoms in mice, mortality rarely occurs (32). In contrast, intraperitoneal injection significantly increased the infection success rate, ensuring the reliability of the experimental outcomes.

### 4.3 Immunoprotective role of nontoxin proteins

In the present study, the Plc, ESBP and YnjE proteins all demonstrated significant immunoprotective effects. Notably, the ESBP extracellular solute-binding protein and the YnjE rhodanese-like domain-containing protein are more likely to participate in metabolic processes than to be directly associated with virulence. The mechanism by which these nontoxin proteins confer protection warrants further investigation.

ESBP extracellular solute-binding protein is processed into a surface-associated lipoprotein and secreted extracellularly via vesicles, where it binds substrates to facilitate the uptake of various solutes, including amino acids, carbohydrates, and metal ions (16, 33). Previous studies have reported that FhuD2, a solute-binding protein in *Staphylococcus aureus*, mediates iron acquisition during invasive infection and that iron is crucial for bacterial biofilm formation (34, 35). Similarly, AdcA, a solute-binding protein in *Streptococcus pneumoniae*, possesses structural and functional features enabling selective zinc uptake, and zinc serves as an essential nutrient for the virulence of many pathogens (36). In *C. perfringens*, Plc is a zinc-dependent toxin, and studies have demonstrated that its virulence and enzymatic activity significantly decrease in zinc-deficient environments but recover upon zinc supplementation (37). Although no studies have confirmed that ESBP in *C. perfringens* is involved in the scavenging of zinc or iron from the environment, this hypothesis could explain why ESBP provides substantial protection against *C. perfringens* infection in mice.

The YnjE rhodanese-like domain-containing protein belongs to an ancient and multifunctional protein superfamily. In many bacterial species, YnjE is involved primarily in sulfate and thiosulfate metabolism (38–40). Owing to the diversity of proteins within this family, a single organism may harbor multiple rhodanese-like superfamily members, each fulfilling distinct physiological roles, including hydrolase activity, signal transduction, and selenium metabolism (41). Given that YnjE likely plays a critical role in fundamental bacterial survival processes, functional impairment—such as antibody-mediated neutralization—could severely disrupt bacterial viability, which may explain why YnjE functions as a protective antigen.

These data suggest that these two nontoxin proteins may promote *C. perfringens* infection via “nutrient uptake-virulence enhancement” and “metabolic hub-survival essentiality” mechanisms. However, further research is needed to clarify their specific mechanisms of action as protective antigens.

### 4.4 Protective antigens in vaccines

This study confirmed that ESBP and YnjE proteins exhibit significant immunoprotective potential. To investigate the levels of specific antibodies induced by these two proteins in mice immunized with commercial vaccines, indirect ELISA assays were performed. The results showed that the peak titers of serum-specific antibodies in groups immunized with recombinant ESBP or recombinant YnjE alone were relatively high. In the challenge experiments, the survival rates of mice in the two groups reached 85% and 90%, respectively, with their body weights recovering rapidly to the initial levels. In contrast, the specific antibody titers against ESBP and YnjE in the Vac3 immunized group were only approximately 1:8,000 and 1:4,000, respectively. Moreover, the survival rate and body weight recovery ability of this group after challenge were significantly lower than those of the groups immunized with recombinant proteins alone (P<0.05).

The above differences suggest that the immunogenicity of ESBP and YnjE proteins in Vac3 was not fully activated. Two main reasons are hypothesized for this observation: first, Vac3 is a whole-bacterial inactivated vaccine containing a variety of antigens in its components. The antigenic competition among different antigens would divert the immune response resources of the body, thereby weakening the specific immune responses against ESBP and YnjE proteins (42, 43). Second, the inactivation and subsequent preparation processes of whole-bacterial vaccines might induce conformational changes in ESBP and YnjE proteins or mask their key antigenic epitopes (44), ultimately reducing the immunogenicity of these two proteins.

It can thus be concluded that the protective advantages exhibited by recombinant ESBP and YnjE proteins in single immunization are essentially attributed to the fact that the immune activity of these two protective antigens is not interfered with by vaccine preparation processes and antigenic competition. In contrast, within the Vac3 system, the immunoprotective potential of these two proteins fails to be fully exerted.

Of note, although Vac1 and Vac3 contained similar Clostridium species, they may differ significantly in the strain type and antigen ratio of *C. perfringens*. This is an interesting observation: different strains naturally vary in antigen expression level and immunogenicity, and differences in antigen component ratio can also directly affect the overall protective efficacy. Collectively, these variations in strain type and antigen ratio suggest that the key protective antigens in the vaccine are likely present within these differential components, and such targets warrant further in-depth exploration and research. This may also be an important reason for the superior protective efficacy of Vac1 over Vac3.

This study revealed that ESBP and YnjE proteins exhibit better immune effects than some vaccines do in a mouse model. However, mice serve as a surrogate model for immunity and infection, and the experimental results obtained in this mouse model need to be further verified in cattle. Continuing to investigate the mechanisms underlying the immunoprotective effects of ESBP and YnjE proteins will aid in understanding the pathogenic mechanisms of *C. perfringens*, and exploring their immunoprotective potential in vaccines will facilitate the enhancement of vaccine immune efficacy.

## 5 Conclusion

The epitopes of the ESBPand YnjE proteins all show high immunogenic potential and are worthy of further study. These proteins demonstrated good immunogenicity and effective resistance to *C. perfringens* infection in challenge tests. Notably, ESBP and YnjE exerted protective efficacy comparable to that of commercial vaccines in murine models, yet their actual protective effects in cattle await further verification in *in vivo* experiments. While ESBP and YnjE possess robust immunogenicity and prominent protective activity, their immunological potential is not fully exploited in some of the commercial inactivated vaccines investigated in this study, likely due to antigen competition and vaccine preparation-related structural changes of the target proteins. Collectively, these findings suggest the application potential of ESBP and YnjE as core candidate antigens and provide a rationale for further investigation into their use in optimized vaccine formulations against *C. perfringens*.

## Data Availability

The original contributions presented in the study are included in the article/**Supplementary Material**. Further inquiries can be directed to the corresponding authors.

## Supplementary material

The supplementary material include Figures S1–S8 and Tables S1–S3, covering the following content: The 18 candidate proteins preliminarily screened by our research team. Preliminary Challenge Protection Assay of SodF Protein. Determination of Median Lethal Dose (LD_50_) and Absolute Lethal Dose (LD_100_) Across Distinct Mouse Strains. Supplementary Information and Clarifications on Commercial Vaccines.
