# Supplementary material for: Protective antigens hidden in the bovine Clostridium vaccine
Source: Front Immunol. 2026 Mar 2;17:1751922. doi: 10.3389/fimmu.2026.1751922 (PMC12989499; doi:10.3389/fimmu.2026.1751922)
Supplement: Supplementary file 1 [file Table1.docx]

Supplementary Material

**Part 1: Workflow for Screening Candidate Antigenic Proteins**


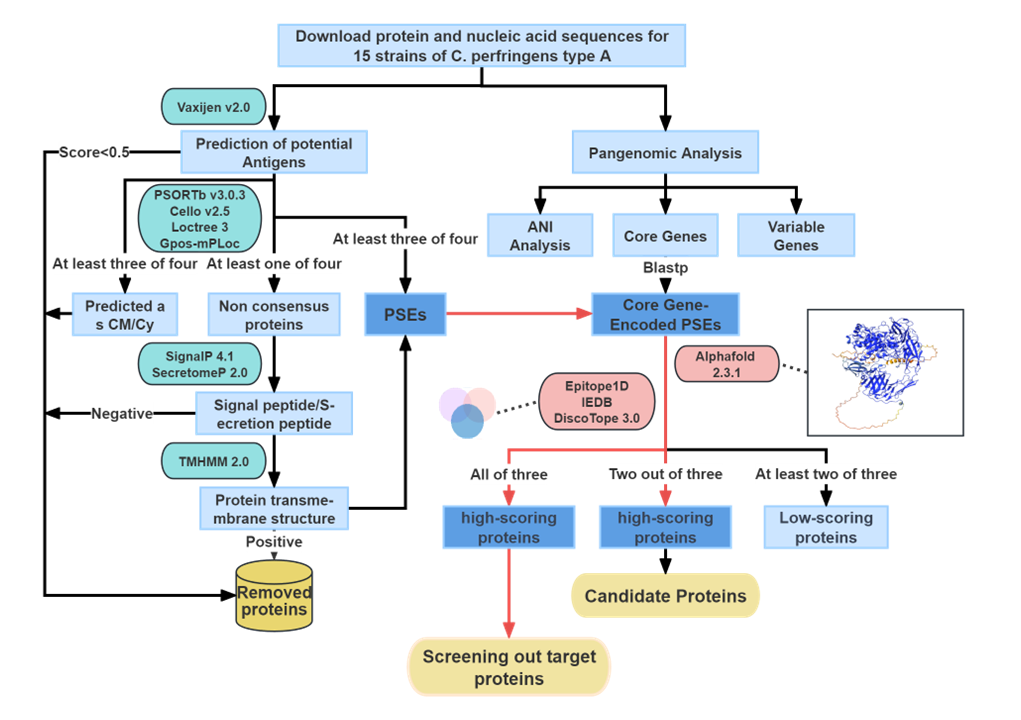


**Figure S1.** Flowchart for antigen protein selection assisted by reverse vaccinology combined with B-cell epitope prediction. VaxiJen categorizes proteins from the *C. perfringens* genome into potential antigens and nonantigens. Potential antigens were localized via the subcellular localization tools pSORTb v3.03, CELLO v2.5, Loctree3, and Gpos-mploc, with those predicted by at least three tools as extracellular or cell wall components defined as potential surface-exposed (PSE) proteins, whereas proteins predicted to be cytoplasmic or membrane-bound were excluded. Proteins with inconsistent subcellular localization predictions were defined as nonconsensus proteins. Nonconsensus proteins were further analyzed using the SignalP, SecretomeP, and TMHMM tools to predict the signal peptides, secretory peptides, and transmembrane structures. Proteins with signal peptides but without transmembrane structures are marked as PSEs; others were excluded. Pangenomic analysis identified core and variable genes. PSEs corresponding to core genes were subjected to B-cell linear and conformational epitope prediction using Epitope1D, IEDB, and DiscoTope, with the top 30 scoring proteins from every prediction being termed high-scoring proteins, and the rest termed low-scoring proteins.

**Part 2: Provenance of the Four Proteins Utilized in This Study**


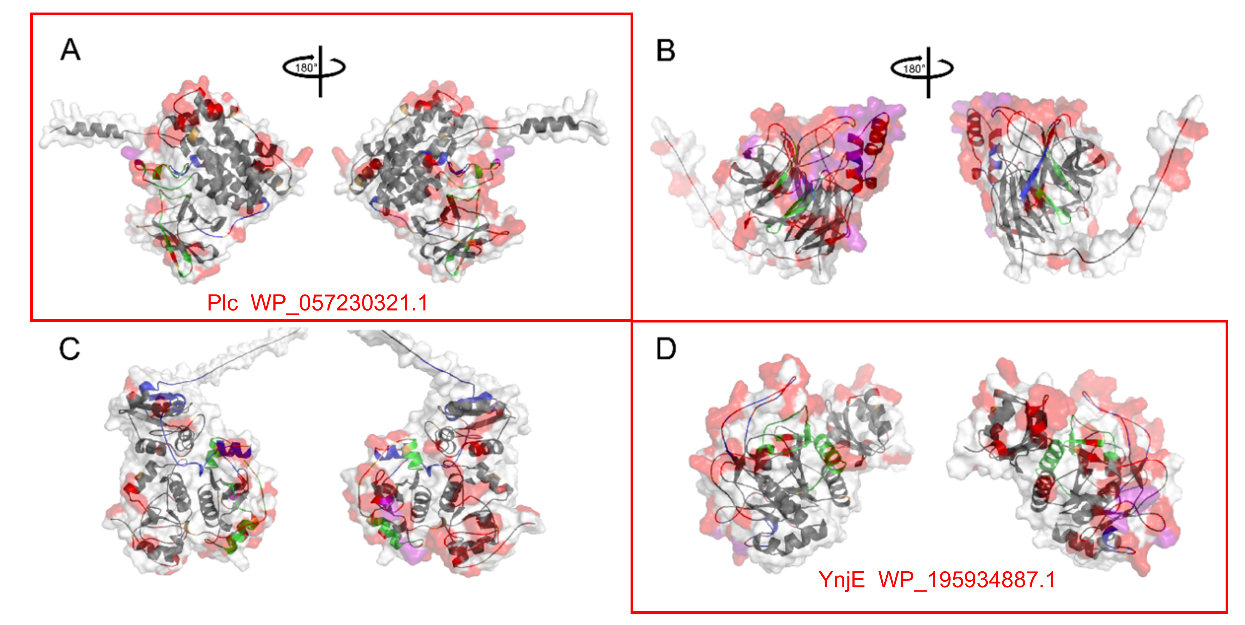


**Figure S2.** 3D map of the target proteins and their B-cell epitopes. (A) Alpha toxin; (B) Cyclically-permuted mutarotase family protein; (C) S8 family serine peptidase; (D) Rhodanese-like domain-containing protein. The illustrations include two structural representations: the surface and the cartoon. On the surface structure, conformational epitopes are colored red and purple, representing score intervals of 0.2-0.4 and 0.4-0.8, respectively. Epitopes within the 0.15-0.2 score range are displayed only on the cartoon structure. In the cartoon structure, blue indicates the two linear epitopes with the highest scores from IEDB, whereas green represents the two linear epitopes with the top scores from Epitope1D. The protein was rotated 180° along the y-axis.


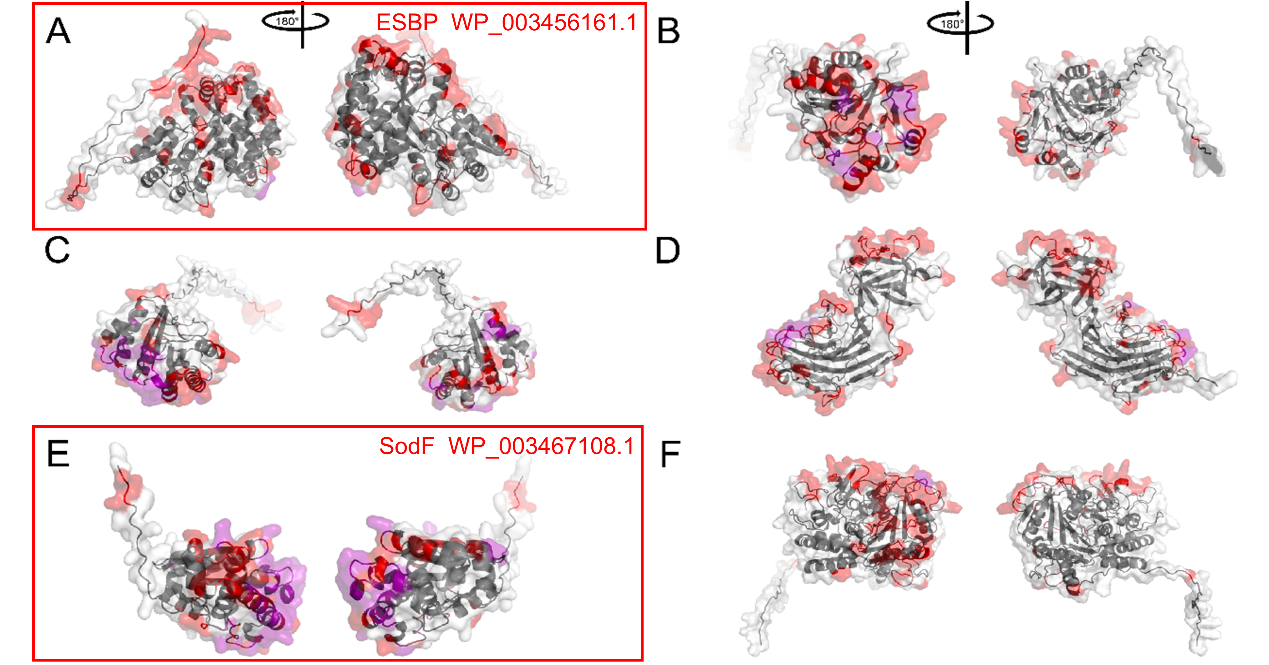


**Figure S3.** 3D map of the Candidate Proteins and its Conformational epitopes. (A) Extracellular solute-binding protein; (B) ComEC/Rec2 family competence protein; (C) Polysaccharide deacetylase family protein; (D) Glycoside hydrolase family 16 protein; (E) Fe-Mn family superoxide dismutase; (F) Clostripain. On the Surface structure, conformational epitopes are colored in red andpurple, representing score intervals of 0.2-0.4 and 0.4-0.8, respectively. The protein was rotated 180° along the y-axis.


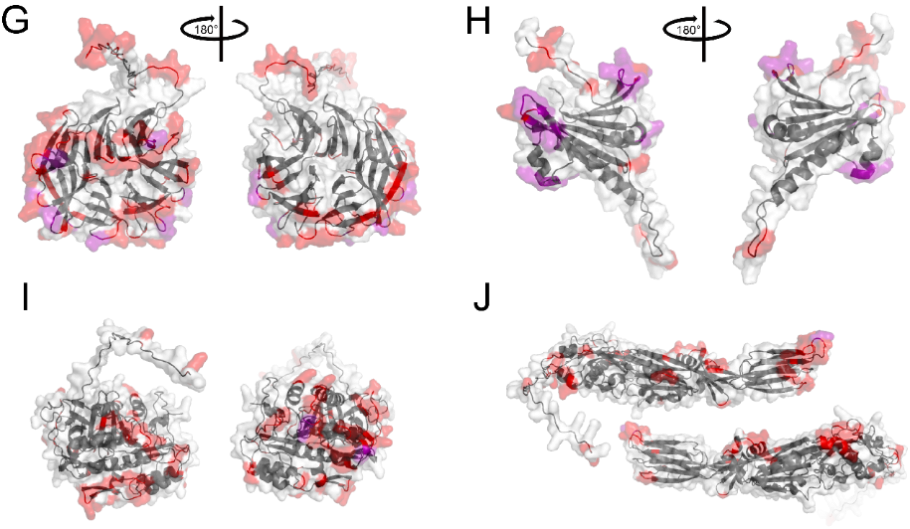


**Figure S4.** 3D map of the Candidate Proteins and its Conformational epitopes.(G) DPP IV N-terminal domain-containing protein; (H) hypothetical protein; (I) Polysaccharide deacetylase family protein; (J) Cholesterol-dependent cytolysin perfringolysin O.


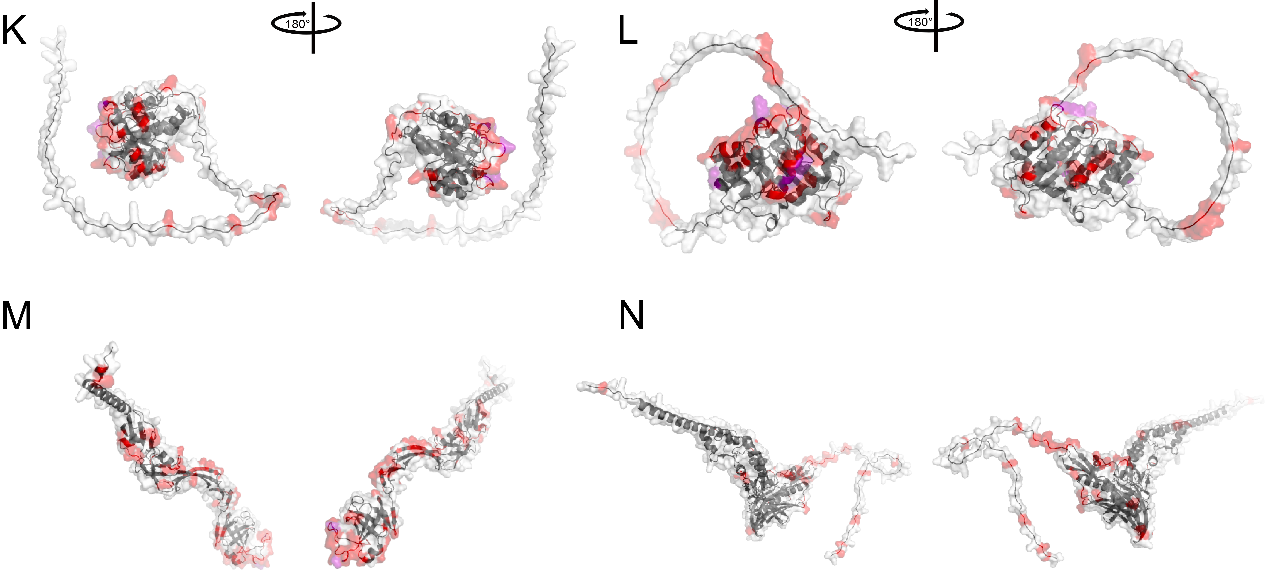


**Figure S5.** 3D map of the Candidate Proteins and its Conformational epitopes. (K) Polysaccharide deacetylase family protein; (L) Polysaccharide deacetylase family protein; (M) 3D domain-containing protein; (N) VanW domain-containing protein.

**Table S1** Detailed prediction information of four proteins in this study

| Abbreviation of  protein name | aa | Vaxijen^a^ | Cellular Localisation | DiscoTope^b^ | Epitope1D^c^ | IEDB^d^ | VF^e^ genes |
| --- | --- | --- | --- | --- | --- | --- | --- |
| ESBP | 419 | 0.520 | Extracellular | 49.2 | 0.640 | 0.653 | - |
| SodF | 227 | 0.515 | Extracellular | 96.1 | 0.637 | 0.627 | - |
| Plc | 398 | 0.517 | Extracellular | 46 | 0.711 | 0.706 | *plc* |
| YjnE | 451 | 0.527 | Extracellular | 46.7 | 0.650 | 0.687 | - |

a The threshold for predicting whether it is an antigen using the Vaxijen tool is 0.4; b The weighted score after prediction of each protein by DiscoTope; c Epitope1D predicts the score of peptide sequences, with a score greater than 0.5 indicating a linear epitope; d The IEDB prediction threshold is also 0.5; e VF is the abbreviation for ‘virulence factor’;

**Part 3: Suboptimal Immunogenicity and Protective Efficacy of SodF Protein in Pilot Experiments**


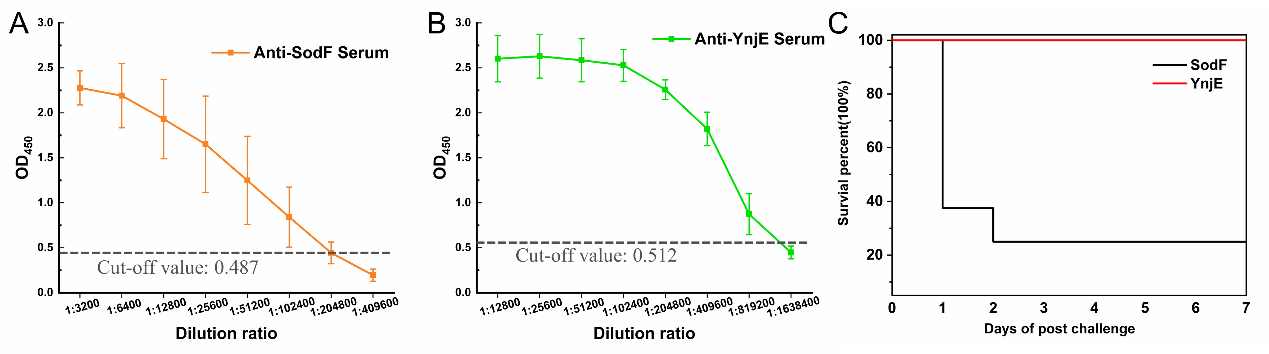


**Figure S6.** Analysis of Antibody Responses and Protective Efficacy of SodF and YnjE Protein Immunization. (A) Serum-specific antibody titers after three immunizations with SodF protein; (B) Serum-specific antibody titers after three immunizations with YnjE protein; (C) Survival curves of SodF- and YnjE-immunized mice challenged with *Clostridium perfringens* ATCC 13124. **Experimental design**: KM mice were randomly divided into SodF group and YnjE group (n=8/group). Immunization was administered via intraperitoneal injection, with a dose of 100μg of antigen protein per mouse, for a total of three doses with a 14 days interval between each immunization. The challenge dose for each mouse is 0.15mL, and the bacterial solution concentration is 3.7 × 10 ⁸ CFU/mL.

**Part 4: Determination of Median Lethal Dose (LD₅₀) and Absolute Lethal Dose (LD₁₀₀) Across Distinct Mouse Strains**

**Methods**

**Preparation of ATCC13124 Bacterial Strain:**

The ATCC13124 bacterial strain was anaerobically cultured until the OD_600_ value reached approximately 0.6. Bacteria were harvested by centrifugation, the supernatant was discarded, and the bacteria were washed twice with PBS. The bacterial pellet was resuspended in PBS and diluted to four different concentrations with OD_600_ values of 0.4, 0.6, 0.8, and 1.0. The four concentration gradients of the bacterial suspension mentioned above were further diluted in series, and 0.1 mL aliquots were plated onto solid Sulfite-Polymyxin-Sulfadiazine (SPS) agar plates, followed by anaerobic incubation at 37°C for 12 hours. After incubation, colonies were enumerated to calculate colony-forming units per milliliter (CFU/mL).

**Calculation of LD_50_ in BALB/c Mice:**

Thirty female BALB/c mice were randomly divided into five groups: a PBS control group and four experimental groups, with six mice in each group. The experimental groups received intraperitoneal injections of bacterial suspensions at OD_600_ values of 0.4, 0.6, 0.8, 1.0, and 1.2 (0.15 mL per mouse), while the control group received 0.15 mL of sterile PBS. The mice were observed continuously for 7 days to monitor clinical symptoms and record mortality rates. The LD_50_ was calculated using the Probit procedure in SPSS 26.0.

**Calculation of LD_50_ and LD_100_ in Kunming Mice:**

The ATCC13124 bacterial strain was anaerobically cultured until the OD_600_ value reached approximately 0.6. Bacteria were collected by centrifugation, washed twice with PBS, and resuspended in PBS. The bacterial suspension was diluted to five different concentrations with OD_600_ values of 0.4, 0.6, 0.8, 1.0, and 1.2. Colony-forming unit (CFU) enumeration was performed identically to the aforementioned protocol, with serial dilution, anaerobic plating on SPS agar, and incubation at 37°C for standardized quantification (CFU/mL). Forty-eight female Kunming mice were randomly divided into six groups: a PBS control group and five experimental groups, with eight mice in each group. The experimental groups received intraperitoneal injections of bacterial suspensions at OD_600_ values of 0.4, 0.6, 0.8, 1.0, and 1.2 (0.15 mL per mouse), while the control group received 0.15 mL of sterile PBS. The mice were observed continuously for 7 days to monitor clinical symptoms and record mortality rates. The LD_50_ was calculated using the Probit procedure in SPSS 26.0. The LD_100_ was estimated based on the SPSS analysis results and the mortality data of the mice.

**Results**

After calculating by the plate colony counting method, the bacterial concentrations corresponding to OD_600_ values of 0.4, 0.6, 0.8, 1.0, and 1.2 were approximately 1.4×10⁶, 7.2×10⁷, 3.7×10⁸, 1.7×10⁹, and 2.5×10¹⁰ CFU/mL, respectively. When infecting BALB/c mice with ATCC13124 strain at these five different concentrations, the mortality of the mice is shown in Table S2. The mortality statistics for KM mice are presented in Table S3.

**Table S2** Statistical results of the amount of bacteria for challenge and the death of BALB/c mouse

| Group | OD_600_ Value | Concentration（CFU/mL） | Number of Mice | Number of Deaths | Mortality Rate (%) |
| --- | --- | --- | --- | --- | --- |
| Experimental Group | 0.4 | 1.4×10^6^ | 6 | 0 | 0% |
|  | 0.6 | 7.2×10^7^ | 6 | 1 | 16.7% |
|  | 0.8 | 3.7×10^8^ | 6 | 4 | 66.7% |
|  | 1.0 | 1.7×10^9^ | 6 | 6 | 100% |
| Control | - | 0 | 6 | 0 | 0 |

**Table S3** Statistical results of the amount of bacteria for challenge and the death of KM mouse

| Group | OD_600_ Value | Concentration（CFU/mL） | Number of Mice | Number of Deaths | Mortality Rate (%) |
| --- | --- | --- | --- | --- | --- |
| Experimental Group | 0.4 | 1.4×10^6^ | 8 | 0 | 0% |
|  | 0.6 | 7.2×10^7^ | 8 | 1 | 12.5% |
|  | 0.8 | 3.7×10^8^ | 8 | 3 | 37.5% |
|  | 1.0 | 1.7×10^9^ | 8 | 7 | 87.5% |
|  | 1.2 | 2.5×10^10^ | 8 | 8 | 100% |
| Control | - | 0 | 8 | 0 | 0% |

The median lethal dose (LD₅₀) was determined by Probit analysis using SPSS 26.0. Prior to analysis, bacterial concentrations (CFU/mL) were log₁₀-transformed. For BALB/c mice, the LD₅₀ was calculated to be 2.068×10⁸ CFU/mL, with a 95% confidence interval of 5.669×10⁷ to 6.296×10⁸ CFU/mL. For Kunming mice, the LD₅₀ was 4.339×10⁸ CFU/mL, with a 95% confidence interval of 1.616×10⁸ to 1.275×10⁹ CFU/mL.

In the experimental observation, a concentration of 2.5×10¹⁰ CFU/mL resulted in 100% mortality (8/8) in Kunming mice.

**Part 5: Supplementary Information and Clarifications on Commercial Vaccines**

**
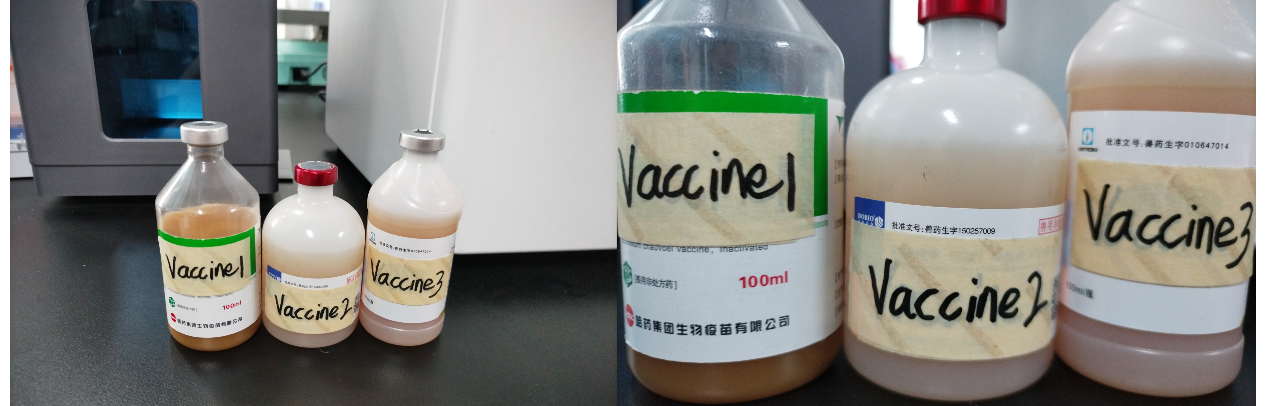
**

**Figure S7.** Pictures of three commercial inactivated vaccines

The three commercial vaccines (Vaccine1-3) in this study are all multivalent inactivated vaccines with veterinary drug production approval numbers. Their antigens include *C. perfringens* type A and other pathogenic clostridial species. As the main biological products for Xinjiang's ruminant immunization program, these vaccines have established extensive cattle and sheep vaccination coverage.


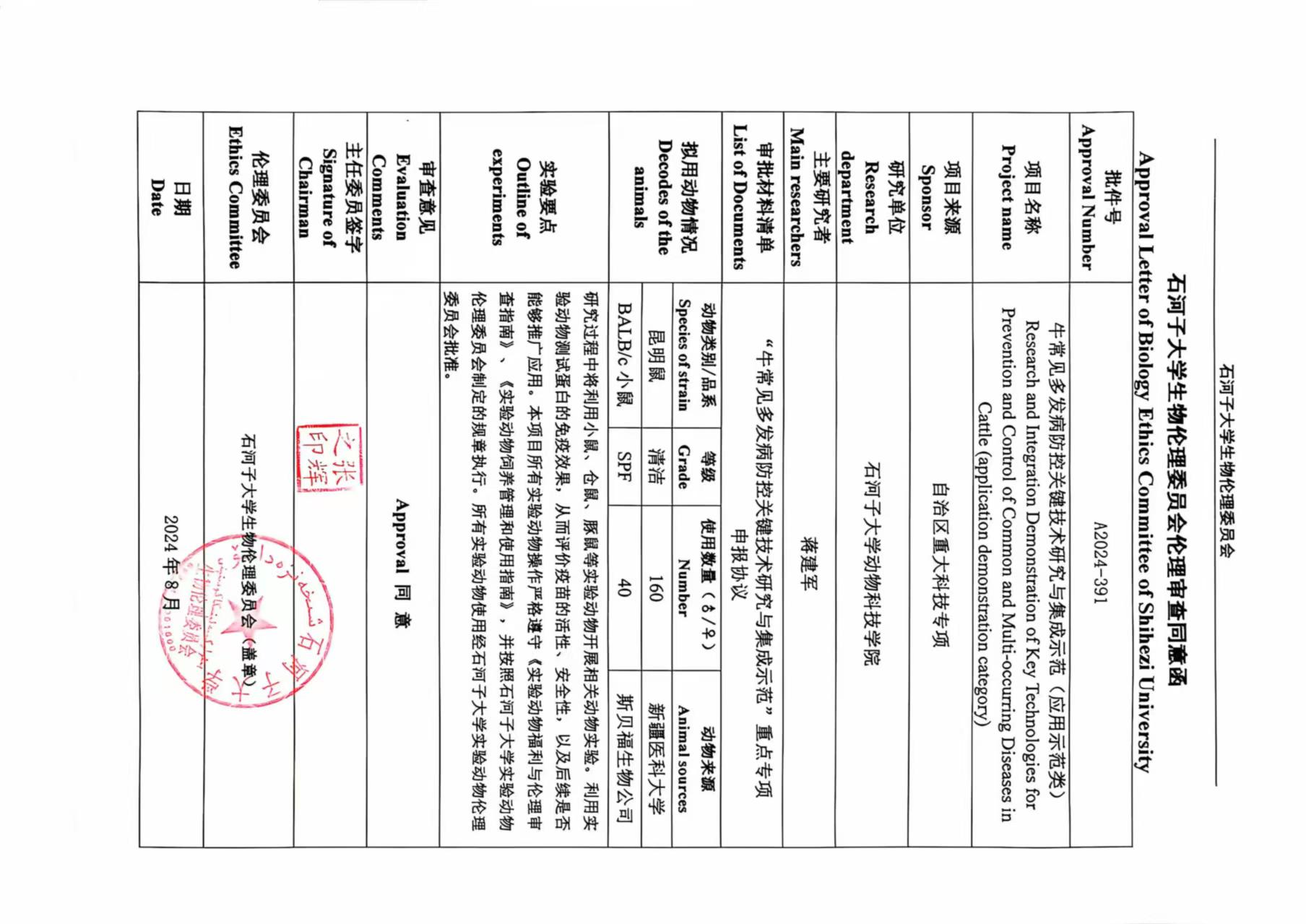


**Figure S8.** Approval letter of biology ethics committee of Shihezi University
